# Supplementary material for: Hypoxia-Inducible Pathway Polymorphisms and Their Role in the Complications of Prematurity
Source: Genes (Basel). 2023 Apr 26;14(5):975. doi: 10.3390/genes14050975 (PMC10218469; doi:10.3390/genes14050975)
Supplement: Supplementary file 1 [file genes-14-00975-s001.zip › genes-2284299-supplementary.pdf]

Supplementary tables

Supplementary Table S1: Reanalysis of a group of infants who meet stricter ACOG criteria for hypoxia.

Parameter	Non-Hypoxic N= 268	Hypoxic N=66	*OR (95%CI); P*
NEC	58 (21.6)	20 (30.3)	1.6 (0.89-2.9), 0.136
**BPD**	**94 (35.1)**	**36 (54.6)**	**2.2 (1.3-3.8), 0.004**
**IVH**	**140 (52.2)**	**52 (78.8)**	**3.4 (1.8-6.4), < 0.0001**
**RDS**	**168 (62.7)**	**52 (78.8)**	**2.2 (1.2-4.2), 0.014**
**ROP**	**158 (59.0)**	**54 (81.8)**	**3.1 (1.6-6.1), 0.0006**
**DWMI**	**16 (5.6)**	**10 (15.5)**	**2.8 (1.2-6.5), 0.013**
Sepsis	60 (22.4)	18 (27.3)	1.3 (0.70-2.4), 0.401
Jaundice	220 (82.1)	58 (87.9)	1.6 (0.71-3.5), 0.259
Death	3 (1.1)	2 (3.0)	2.8 (0.45-17.0), 0.252

Supplementary Table S2. Relationship between *HIF1A* SNPs and the presence of comorbidities of prematurity or hypoxia at birth in premature infants.

**Genotype**	**NEC**		**BPD**		**IVH**		**RDS**		**ROP proliferative**		**DWMI**		**Hypoxic at birth**	
	No n=256	Yes n=78	No n=204	Yes n=130	No n=142	Yes n=192	No n=114	Yes n=220	No n=235	Yes n=99	No n=308	Yes n=26	No n=180	Yes n=154
***HIF1A* rs11549465**														
**CC**	212 (82.8)	73 (93.6)	179 (87.8)	106 (81.5)	128 (90.1)	157 (81.8)	100 (87.7)	185 (84.1)	207 (88.1)	78 (83.5)	265 (86.0)	20 (76.9)	156 (86.7)	129 (83.8)
**CT**	43 (16.8)	4 (5.1)	24 (11.8)	23 (17.7)	14 (9.9)	33 (17.2)	12 (10.5)	35 (15.9)	27 (11.5)	20 (15.6)	42 (13.6)	5 (19.2)	23 (12.8)	24 (15.6)
**TT**	1 (0.4)	1 (1.3)	1 (0.5)	1 (0.8)	0 (0.0)	2 (1.0)	2 (1.8)	0 (0.0)	1 (0.43)	1 (0.9)	1 (0.32)	1 (3.9)	1 (0.6)	1 (0.7)
MAF	0.088	0.038[HWE]	0.064	0.096	0.049	0.096	0.070 [HWE]	0.080	0.062	0.111	0.071	0.135	0.069	0.084
T allele; OR (95%CI); P	**0.42 (0.17-0.99), 0.042**		1.6 (0.88-2.8), 0.124		**2.1 (1.1-3.9), 0.024**		1.2 (0.62-2.1), 0.665		**1.9 (1.1-3.4), 0.028**		2.0 (0.86-4.8), 0.107		1.2 (0.7-2.2), 0.468	
CT+TT vs CC: OR (95%CI); P	**0.33 (0.13-0.86), 0.019**		1.6 (0.88-3.0), 0.118		**2.0 (1.1-4.0), 0.033**		1.3 (0.69-2.6), 0.374		**2.0 (1.1-3.7), 0.041**		1.8 (0.7-4.9), 0.207		1.3 (0.69-2.3), 0.455	
***HIF1A* rs11549467**														
**GG**	242 (94.5)	74 (94.9)	193 (94.6)	123 (95.0)	134 (94.4)	182 (94.8)	103 (90.4)	213 (96.8)	221 (94.0)	95 (96.0)	290 (94.2)	26 (100.0)	172 (95.6)	144 (93.5)
**GA**	14 (5.5)	4 (5.1)	11 (5.4)	7 (5.0)	8 (5.6)	10 (5.2)	11 (9.7)	7 (3.2)	14 (6.0)	4 (4.0)	18 (5.8)	0 (0.0)	8 (4.4)	10 (6.5)
MAF	0.027	0.026	0.027	0.027	0.028	0.026	0.048	0.016	0.030	0.020	0.029	0.000 [HWE]	0.022	0.032
A allele, OR (95%CI); P	0.93 (0.30-2.9), 1.00		0.10 (0.38-2.6), 0.998		0.92 (0.36-2.4), 0.867		**0.32 (0.12-0.83), 0.021**		0.67 (0.22-2.1), 0.607		0.31 (0.02-5.2), 0.386		1.5 (0.58-3.8),0.415	
AG+AA vs GG: OR (95%CI); P	0.93 (0.30-2.9), 0.907		0.89 (0.38-2.6), 0.998		0.92 (0.35-2.4), 0.865		**0.31 (0.12-0.82), 0.020**		0.66 (0.21-2.1), 0.602		0.03 (0.02-5.1), 0.379		1.5 (0.57-3.9),0.471	

Abbreviations and symbols: *BPD*, bronchopulmonary dysplasia; *DWMI*, diffuse white matter injury; *IVH*, intraventricular hemorrhage; *RDS*, respiratory distress syndrome; *ROP*, retinopathy of prematurity, and *NEC*, necrotizing enterocolitis.

Supplementary Table S3. Relationship between *HIF1A* SNPs and the presence of comorbidities of prematurity in relation to hypoxia at birth.

**Genotype**	**NEC**		**BPD**		**IVH**		**RDS**		**ROP proliferative**		**DWMI**	
	No	Yes	No	Yes	No	Yes	No	Yes	No	Yes	No	Yes
***HIF1A* rs11549465**												
**Nonhypoxic; n=180**	n=150	n=30	n=140	n=40	n=101	n=79	n=73	n=107	n=143	n=37	n=171	n=9
**CC**	127 (84.7)	29 (96.7)	122 (87.1)	34 (85.0)	91 (90.1)	65 (82.3)	64 (87,7)	92 (86.0)	128 (89.5)	28 (75.7)	147 (86.0)	9 (100.0)
**CT**	22 (14.7)	1 (3.3)	17 (12.1)	6 (15.0)	10 (9.9)	13 (16.5)	8 (11,0)	15 (14.0)	14 (9.8)	9 (24.3)	23 (13.5)	0 (0.0)
**TT**	1 (0.7)	0 (0.0)	1 (0.7)	0 (0.0)	0 (0.0)	1 (1.3)	1 (1,4)	0 (0.0)	1 (0.7)	0 (0.0)	1 (0.6)	0 (0.0)
**MAF**	0.080	0.017	0.068	0.075	0.050	0.095	0,068	0.070	0.056	0.112	0.073	0.000
OR (95%CI), P	0.19 (0.03-1.5), 0.096		1.05 (0.47-2.4), 0.904		2.0 (0.88-4.6), 0.099		1.0 (0.45-2.3), 0.953		**2.3 (1.0-5.5), 0.048**		0.34 (0.02-5.8), 0.625	
**Hypoxic; n=154**	n=106	n=48	n=64	n=90	n=41	n=113	n=41	n=113	n=92	n=62	n=137	n=17
**CC**	85 (80.2)	44 (91.7)	57 (89.1)	72 (80.0)	37 (90.2)	92 (81.4)	36 (87.8)	93 (82.3)	79 (85.9)	50 (80.6)	118 (86.1)	11 (64.7)
**CT**	21 (19.8)	3 (6.3)	7 (10.9)	17 (18.9)	4 (9.8)	20 (17.7)	4 (9.8)	20 (17.7)	13 (14.1)	11 (17.7)	19 (13.9)	5 (29.4)
**TT**	0 (0.0)	1 (2.1)	0 (0.0)	1 (1.1)	0 (0.0)	1 (0.88)	1 (2.4)	0 (0.0)	0 (0.0)	1 (1.6)	0 (0.0)	1 (5.9)
**MAF**	0.099	0.052	0.055	0.106	0.049	0.097	0.073	0.088	0.071	0.105	0.069	0.206
OR (95%CI), P	0.5 (0.19-1.4), 0.192		2.0 (0.83-5.0), 0.146		2.1 (0.70-6.3), 0.246		1.2 (0.48-3.2), 0.818		1.5 (0.96-3.4), 0.303		**3.5 (1.3-9.0), 0.015**	
***HIF1A* rs11549467**												
**Nonhypoxic; n=180**	n=150	n=30	n=140	n=40	n=101	n=79	n=73	n=107	n=143	n=37	n=171	n=9
**GG**	144 (96.0)	28 (93.3)	134 (95.7)	38 (95.5)	96 (95.1)	76 (96.2)	67 (91.8)	105 (98.1)	135 (94.4)	37 (100.0)	163 (95.3)	9 (100.0)
**GA**	6 (4.0)	2 (6.7)	6 (4.3)	2 (5.0)	5 (5.0)	3 (3.8)	6 (8.2)	2 (1.9)	8 (5.6)	0 (0.0)	8 (4.7)	0 (0.0)
**MAF**	0.020	0.033	0.021	0.025	0.025	0.019	0.041	0.009	0.028	0.000	0.023	0.000
OR (95%CI), P	1.7 (0.33-8.6), 0.625		1,2 (0,23-5,9); 1.000		0.76 (0.18-3.2), 1.000		0.22 (0.04-1.1), 0.066		0.22 (0.01-3.9), 0.369		1.0 (0.05-19.0), 1.000	
**Hypoxic; n=154**	n=106	n=48	n=64	n=90	n=41	n=113	n=41	n=113	n=92	n=62	n=137	n=17
**GG**	98 (92.5)	46 (95.8)	59 (92.2)	85 (94.4)	38 (92.7)	83 (93.8)	36 (87.8)	108 (95.6)	86 (93.5)	58 (93.5)	127 (92.7)	17 (100.0)
**GA**	8 (7.5)	2 (4.2)	5 (7.8)	5 (5.6)	3 (7.3)	7 (6.2)	5 (12.2)	5 (4.2)	6 (6.5)	4 (6.5)	10 (7.3)	0 (0.0)
**MAF**	0.038	0.021	0.039	0.028	0.037	0.031	0.061	0.022	0.000	0.031	0.029	0.000
OR (95%CI), P	0.53 (0.11-2.6), 0.438		0,70 (0,20-2,5), 0,746		0.84 (0.21-3.3), 0.729		0.35 (0.10-1.2), 0.138		0.99 (0.27-3.6), 1.000		0.37 (0.02-6.4), 0.609	

Abbreviations and symbols: *BPD*, bronchopulmonary dysplasia; *DWMI*, diffuse white matter injury; *IVH*, intraventricular hemorrhage; *RDS*, respiratory distress syndrome; *ROP*, retinopathy of prematurity, and *NEC*, necrotizing enterocolitis.

Supplementary Table S4. Relationship between *VEGFA* SNPs and the presence of comorbidities of prematurity or hypoxia at birth in premature infants.

**Genotype**	**NEC**		**BPD**		**IVH**		**RDS**		**ROP proliferative**		**DWMI**		**Hypoxic at birth**	
	No n=256	Yes n=78	No n=204	Yes n=130	No n=142	Yes n=192	No n=114	Yes n=220	No n=235	Yes n=99	No n=308	Yes n=26	No n=180	Yes n=154
***VEGFA* rs2010963**														
**GG**	95 (37.1)	34 (43.6)	84 (41.2)	45 (34.6)	47 (33.1)	82 (42.7)	43 (37.7)	86 (39.1)	85 (36.2)	44 (44.4)	118 (38.3)	11 (42.3)	60 (33.3)	69 (44.8)
**GC**	116 (45.3)	27 (34.6)	85 (41.7)	58 (44.6)	66 (46.5)	77 (40.1)	53 (46.5)	90 (40.9)	106 (45.1)	37 (37.4)	132 (42.9)	11 (42.3)	85 (47.2)	58 (37.7)
**CC**	45 (17.6)	17 (21.8)	35 (17.2)	27 (20.8)	29 (20.4)	33 (17.2)	18 (15.8)	44 (20.0)	44 (18.7)	18 (18.2)	58 (18.8)	4 (15.4)	35 (19.4)	27 (17.5)
MAF	0.402	0.391	0.380	0.431	0.437	0.372	0.390	0.405	0.413	0.369	0.403	0.365	0.431	0.364
C allele, OR (95%CI); P	0.95 (0.66-1.4), 0.801		1.2 (0.90-1.7), 0.191		0.77 (0.56-1.0), 0.094		1.1 (0.76-1.5), 0.723		0.83 (0.59-1.2), 0.288		0.85 (0.47-1.5), 0.599		0.76 (0.55-1.0), 0.078	
***VEGFA* rs833061**														
**CC**	124 (48.4)	40 (51.3)	107 (52.5)	57 (43.8)	74 (52.1)	90 (46.9)	58 (50.9)	106 (48.2)	117 (49.8)	47 (47.5)	154 (50.0)	10 (38.5)	85 (47.2)	79 (51.2)
**CT**	118 (46.1)	32 (41.0)	88 (43.1)	62 (47.7)	61 (43)	89 (46.4)	50 (43.9)	100 (45.5)	105 (44.7)	45 (45.5)	135 (43.8)	15 (57.7)	81 (45.0)	69 (44.8)
**TT**	14 (5.5)	6 (7.7)	9 (4.4)	11 (8.5)	7 (4.9)	13 (6.8)	6 (5.3)	14 (6.4)	13 (5.5)	7 (7.1)	19 (6.2)	1 (3.8)	14 (7.8)	6 (3.9)
MAF	0.285	0.282	0.260	0.323	0.264	0.299	0.272	0.291	0.279	0.298	0.281	0.327	0.303	0.263
T allele; OR (95%CI); P	0.98 (0.66-1.5), 0.940		1.4 (0.97-1.9), 0.077		1.2 (0.85-1.7). 0.316		1.1 (0.77-1.6), 0.606		1.1 (0.76-1.6), 0.614		1.2 (0.68-2.3), 0.479		0.82 (0.59-1.2), 0.256	

Abbreviations and symbols: *BPD*, bronchopulmonary dysplasia; *DWMI*, diffuse white matter injury; *IVH*, intraventricular hemorrhage; *RDS*, respiratory distress syndrome; *ROP*, retinopathy of prematurity, and *NEC*, necrotizing enterocolitis.

Supplementary Table S5. Multivariate statistical analysis to assess the independent effect of *HIF1A* genotype on the development of comorbidities of prematurity.

	Statistical analysis: OR (95%CI), *P*				
	NEC	IVH	RDS	DWMI	ROP_p
**Model 1 all major risk factors**					
Gestational age <28 wk.	**3.1 (1.6-5.8), 0.0007***	**2.5 (1.4-4.3), 0.001***	0.56 (1.20-2.1), 0.343	0.77 (0.25-2.4), 0.651	**8.3 (4.3-15.8), <0.0001***
Body weight <1000 g	**4.1 (2.1-7.8), <0.0001***	**2.5 (1.4-4.3), 0.001***	**2.5 (1.4-4.5), 0.002***	1.9 (0.69-5.5), 0.208	**3.0 (1.6-5.6), 0.0005***
Male sex	1.2 (0.7-2.2), 0.477	1.5 (0.95-2.5), 0.083	1.4 (0.88-2.3), 0.149	1.4 (0.61-3.4), 0.407	1.3 (0.75-2.4), 0.321
Oxygen supply [1 day]	NA	NA	NA	**1.02 (1.01-1.04), 0.011***	NA
rs11549465CT+TT	**0.19 (0.07-0.54), 0.002***	1.6 (0.80-3.4), 0.171	1.1 (0.55-2.3), 0.753	NA	1.3 (0.63-2.9), 0.445
rs11549467GA+AA	0.57 (0.16-2.0), 0.369	0.79 (0.28-2.2), 0.652	**0.24 (0.09-0.68), 0.007***	NA	0.47 (0.13-1.7), 0.258
rs11549465CT+TT and hypoxia	NA	NA	NA	**4.0 (1.2-12.9), 0.020**	NA
Model summary					
OR	NE	4.7	3.4	NE	11.5
Overall accuracy (%)	NE	68.9	67.1	NE	79.9
**Model 2 statistically significant risk factors**					
Gestational age <28 wk.	**3.1 (1.6-5.8), 0.0008***	**2.5 (1.4-4.3), 0.001***	NA	NA	**8.6 (4.6-16.4), <0.0001***
Body weight <1000 g	**3.9 (2.0-7.4), <0.0001***	**2.5 (1.4-4.3), 0.001***	**2.6 (1.6-4.4), 0.002***	NA	**2.7 (1.5-5.1), 0.001***
Oxygen supply [1 day]	NA	NA	NA	**1.02 (1.01-1.03), 0.0003***	NA
rs11549465CT+TT	**0.21 (0.08-0.58), 0.003***	NA	NA	NA	NA
rs11549467GA+AA	NA	NA	**0.25 (0.09-0.68), 0.007***	NA	NA
rs11549465CT+TT and hypoxia	NA	NA	NA	**4.5 (1.4-14.1), 0.009***	NA
Model summary					
OR	7.3	4,7	3,2	12.3	12.2
Overall accuracy (%)	78.4	68,9	67,1	92.2	80.2

Abbreviations and symbols: *DWM*, diffuse white matter injury; *IVH*, intraventricular hemorrhage; RDS, respiratory distress syndrome; *ROP_p*, proliferative retinopathy of prematurity, and *NEC*, necrotizing enterocolitis; *NA*, not analyzed; *NE*, not estimable; Overall accuracy-percent correct classification (PCC)

Statistical analysis: *- met the criteria of the Bonferroni correction for multiple comparisons
